# Differential effect of body mass index on the incidence of diabetes and diabetic retinopathy in two Asian populations

**DOI:** 10.1038/s41387-018-0018-0

**Published:** 2018-03-07

**Authors:** Joel Chee Yee Chan, Miao Li Chee, Nicholas Yi Qiang Tan, Ching-Yu Cheng, Tien Yin Wong, Charumathi Sabanayagam

**Affiliations:** 1 Singapore Eye Research Institute, Singapore National Eye Centre, The Academia, 20 College Road, Discovery Tower Level 6, Singapore, 169856 Singapore; 2 Department of Ophthalmology, Yong Loo Lin School of Medicine, National University of Singapore, Singapore, Singapore; 3 Ophthalmology and Visual Sciences Academic Clinical Program, Duke-NUS Medical School, Singapore, Singapore; 4 Centre for Quantitative Medicine, Duke-NUS Medical School, Singapore, Singapore

## Abstract

**Aims:**

To examine the association of body mass index (BMI) with the incidence of diabetes mellitus (DM) and diabetic retinopathy (DR) in Asians.

**Methods:**

We analysed data from 4101 adults (Malay, *n* = 1901 and Indian, *n* = 2200) who participated in the baseline (2004–2009) and 6-year follow-up (2011–2015) of two independent population-based studies with similar methodology in Singapore. BMI was categorised into normal (<25 kg/m^2^), overweight (25–29.9 kg/m^2^) and obese (≥30 kg/m^2^). DM was diagnosed as random plasma glucose ≥200 mg/dL, HbA_1c_ ≥6.5% or self-reported physician diagnosed DM. DR was assessed from retinal photographs graded using a standard protocol. The associations of baseline BMI with incident DM and DR was examined using multivariable poisson regression models adjusting for potential confounders including duration of DM, family history of DM and HbA_1c_.

**Results:**

The incidence of DM was 12.8% and among 1586 participants with DM, the incidence of DR was 17.6% over a median follow-up period of 6.2 years. Compared to those with BMI < 25, the relative risk (95% confidence interval) of incident DM was 1.77 (1.36–2.29) for overweight and 2.01 (1.50–2.71) for obese (*p* trend < 0.001). Relative risk of DR was 0.80 (0.59–1.09) for overweight and 0.60 (0.39–0.92) for obese (*p* trend = 0.02). In analyses stratified by ethnicity, similar pattern of associations with DM and DR were observed in both ethnicities.

**Conclusion:**

Our results suggest that, overweight and obesity increased the 6-year risk of DM but decreased the 6-year risk of DR in these Asian populations.

## Introduction

Diabetes mellitus (DM) is projected to increase to 592 million in 2035, with Asia having the highest number of individuals with DM globally and importantly, with the prevalence increasing at a much faster rate than in Western countries^1^. Diabetic retinopathy (DR), a major complication of DM, accounts for 5% of all blindness affecting up to 2 million people worldwide yet up to 90% of blindness resulting from DR is preventable if adequate screening and evidence-based care for DM were to be implemented^2^. The classic risk factors for DR include longer duration of DM, poor glycemic control reflected by higher glycated haemoglobin (HbA_1c_) levels and hypertension^3–6^.

While obesity is an established risk factor for DM^7^, and has been postulated to be also a risk factor for the development of DR^8^, the association of body mass index (BMI) with DR in previous studies have not shown consistent results. Furthermore, Asians have been shown to have different associations between BMI, percentage body fats and health risks such as cardiovascular disease as compared to Western populations^9–11^. Previous research suggests that BMI could influence DR differently in Asian and Western populations^8, 12–20^. Thus, while Western studies have showed higher BMI and obesity is related to DR^8, 11, 12^ the majority of studies in Asia have shown that lower BMI is related to DR^13–17, 19^. Importantly, few studies have been prospective in nature^10, 11^ and the relationship between baseline BMI and the subsequent risk of DR is unclear.

To address these gaps, in two we examined the relationship of baseline BMI with 6-year risk of DM and the 6-year risk of DR in Asian adults with DM in Singapore.

## Methods

### Study participants

Data for the current study was derived from two prospective cohort studies: the Singapore Malay Eye Study (SiMES, 2004–2006, SiMES-2, 2011–2013), and the Singapore Indian Eye Study (SINDI, 2007–2009, SINDI-2, 2012–2015). Details of the design of the above studies, sampling plan and methodology have been extensively published elsewhere^21, 22^.

In brief, a total of 3280 of the 4168 eligible individuals participated at baseline in SiMES (78.7% response rate), and 3400 of the 4497 eligible individuals participated at baseline in SINDI (75.6% response rate). Of the 3280 participants in the SiMES cohort, 1052 had DM and 295 had DR at baseline; of the 3400 participants in the SINDI cohort, 1320 had DM and 418 had DR at baseline. In the respective follow-up studies (median follow-up period of 6.2 years), a participant from the original studies was considered to be subsequently ineligible if the he or she had moved from the address of residence, had not lived in the residence in the past 6 months, or was terminally ill or deceased. Thus, after excluding 644 and 486 ineligible SiMES and SINDI participants respectively, 1901 of the 2636 (72.1% response rate) eligible SiMES participants, and 2200 of the 2914 (75.5% response rate) eligible SINDI participants attended the follow-up studies^23, 24^.

**Fig. 1:**
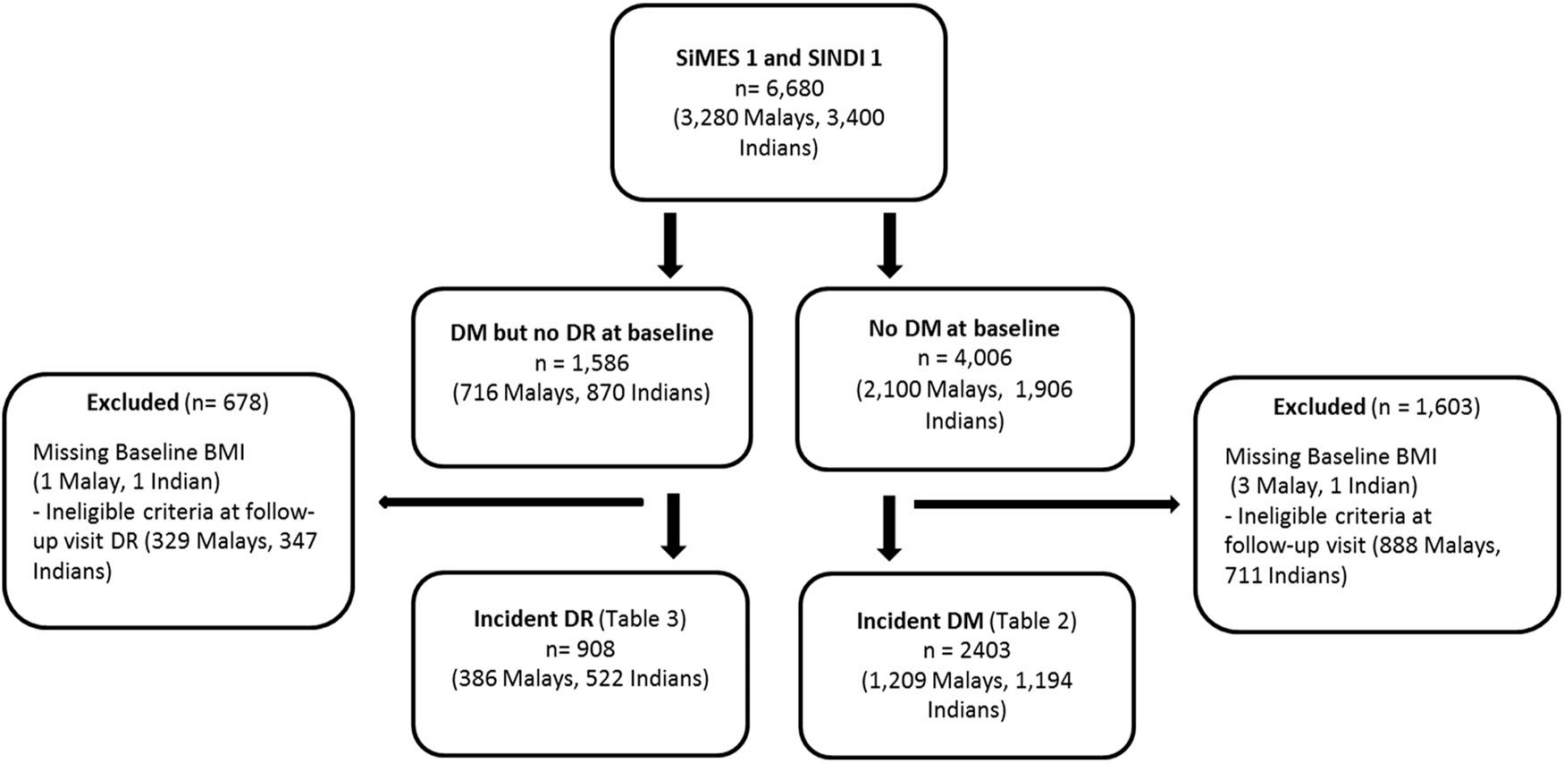
Flowchart for the inclusion and exclusion of participants

For the current analyses, we included only participants who attended both baseline and follow-up (1901 Malays and 2200 Indians). Since the methodology for SiMES and SINDI were similar, we combined data from the two cohorts for the main analysis. For outcome, ‘Incidence of DM’, we excluded 2372 participants with prevalent DM at baseline, and 4 participants with missing data on key variables; this left us with 2403 participants without DM for inclusion (Fig. 1). For outcome, ‘Incidence of DR’, we excluded 713 participants with prevalent DR at baseline, and 2 participants with missing data; this left us with 908 participants with DM eligible for inclusion in the analysis (Fig. 1). For outcome, ‘Incident DM’, our sample size of 2403 and 308 number of cases had 80% power to detect a RR of minimum 1.55 comparing normal versus overweight and 1.76 comparing normal versus obese respectively. For continuous BMI, we had 80% power to detect RR of minimum 1.03 for incident DM per unit increase in BMI. For outcome, ‘Incident DR, our sample size of 908 and 160 number of cases had 80% power to detect a RR of minimum 0.67 comparing normal versus overweight and 0.63 comparing normal versus obese respectively. For continuous BMI, we had 80% power to detect RR of minimum 0.93 for incident DR per unit increase in BMI. The above power calculations were done based on significance level of 0.05.

The study adhered to the Declaration of Helsinki, ethics approval was obtained from the SingHealth Centralised Institutional Review Board, and written informed consent was obtained from all participants.

### Assessment and definition of DM, BMI and other risk factors

All participants underwent a standardised questionnaire, clinical examination and the collection of blood samples to assess for non-fasting glucose, HbA_1c_ and lipid profile. Questionnaire data included ethnicity, education, income, personal history of DM, hypertension, family history of DM, smoking history, use of medications and duration of DM. Income level was categorised into two groups (<Singapore dollar [S$]2000, and ≥S$2000); level of education was also categorised into two groups (primary school education and below and above primary school education).

Height was measured in centimetres using a wall-mounted measuring tape and weight was measured in kilograms using a digital scale (SECA, model 7,822,321,009; Vogel and Halke, Germany). BMI was defined as the weight in kilograms divided by the square of height in metres (kg/m^2^). BMI was categorised into underweight (BMI < 18.5 kg/m^2^), normal (18.5 ≤ BMI < 25 kg/m^2^), overweight (25 ≤ BMI < 30 kg/m^2^) and obese (≥30 kg/m^2^) according to BMI cut-off points^9^. BP measurements were taken using a digital automatic blood pressure monitor after the participant was seated for at least 5 min and an average of two measurements were taken as the blood pressure value for that individual. Hypertension was defined as a systolic BP of 140 mmHg or greater, diastolic BP of 90 mmHg or greater, or use of antihypertensive medication^25^. A standardised questionnaire was conducted for all participants in English or their mother tongue (Malay or Tamil) by trained interviewers. DM was defined as self-reported physician’s diagnosis, use of insulin, use of oral hypoglycaemia medications or random plasma glucose ≥200 mg/dL or HbA_1c_ ≥ 6.5% (48 mmol/mol)^26, 27^. We defined type 1 DM as those who developed DM before the age of 30 years. For the incident analysis, since we excluded those with DM including those who developed DM before the age of 30 years (*n* = 8), we assume that those who developed DM at follow-up fall under type 2 DM, i.e., age of onset after 30 years. Incident DM was defined as onset of DM at the follow-up among those who were free of DM at baseline.

### Retinal photography and diabetic retinopathy assessment

Two-field retinal photography was taken after pupil dilation according to the Early Treatment for DR Study (ETDRS) protocol using a 45° nonmydriatic digital retinal camera (Canon CR-DGI with a 10D/20D SLR backing, Canon, Japan)^27^. Retinal images were graded for DR by trained graders masked to participant characteristics. Lesions considered to be characteristic of DR were: microaneurysms, haemorrhages, cotton wool spots, intraretinal microvascular abnormalities, hard exudates, venous beading and new vessels were seen^28^. For each eye, DR severity score was assigned based on the modified Airlie House Classification^28^ into none (ETDRS levels 10–15), minimal non-proliferative (NPDR, level 15–20), mild NPDR (level 35), moderate NPDR (level 43–47), severe NPDR (level 53), or proliferative DR (PDR, score >60)^28^. Based on the worse eye score, any DR was defined as a severity level of 15 and above and vision-threatening DR (VTDR) as presence of severe NPDR, PDR, or clinically significant diabetic macular edema (DME)^28^.

Incident DR was defined as severity level >15 and incident VTDR as those who developed VTDR at the follow-up visit among those who were free of VTDR at baseline (which includes minimal, mild, moderate NPDR).

### Statistical analysis

Analyses were conducted using Stata Version 14.0 (StataCorp LP, College Station, TX) separately for the two main outcomes: incident DM and incident DR. For the present analysis, since the prevalence of being underweight was low (3.1%), we combined underweight and normal as one category and defined normal as BMI < 25 kg/m^2^. We compared baseline characteristics of participants stratified by those 1) with and without DM and 2) with and without DR using the Chi-square test or the independent *t*-test as appropriate for the variable. We examined the associations of baseline BMI (categorical, as well as continuous, per SD increment) with the two outcomes using two poisson regression models with robust variance:^29^ 1) an age, gender and ethnicity-adjusted model; 2) a multivariable model adjusting for other covariates as well, such as income and education, current smoking, family history of DM, HbA_1c_, total cholesterol, HDL cholesterol, systolic BP and diabetes duration. *P*-trend was calculated using BMI categories as an ordinal variable. To examine for consistency of the association between BMI and incident DM or incident DR, we performed subgroup analyses stratified by gender and ethnicity. We examined interactions by gender and ethnicity by including cross-product interaction terms in the corresponding multivariable models. Additionally, the associations of BMI and incident VTDR was also examined with multivariable poisson regression models with robust variance^29^. In supplementary analyses, firstly, we excluded underweight participants (*n* = 84) from the analyses and repeated the main multivariable models; secondly, we examined the associations using WHO Asian BMI categories (BMI < 23, 23–27.5, ≥27.5 for normal, overweight and obese)^9^. In the current study, since only 33 participants (3.7%) were under treatment with fenofibrate and 16 participants were on insulin (1.8%), we did not assess the influence of these drugs in the onset of DR.

## Results

Baseline characteristics of the study population are presented in Table 1. Compared to participants with no DM, those with DM were more likely to be Indians, and less likely to be current smokers; had higher prevalence of hypertension and use of anti-hypertensive medications, family history of DM; had higher levels of HbA_1c_, BMI, systolic and diastolic BP and lower HDL cholesterol level. Compared to participants with no DR, those with DR were younger, more likely to be men and current smokers, had higher prevalence of antidiabetic medication use, higher levels of HbA_1c_, diastolic BP and total cholesterol but lower prevalence of anti-hypertensive medication use and had lower levels of income and BMI.

**Table 1:** Baseline characteristics of participants with and without incident diabetes and with and without incident DR

	Incident DM (*n* = 308)	No incident DM (*n* = 2095)	*P-value	Incident DR (*n* = 160)	No incident DR (*n* = 748)	*P-value
Age (years)	54.5 (8.9)	55.1 (9.4)	0.31	57.4 (9.6)	58.9 (9.2)	0.07
Gender, men (%)	145 (47.1)	1008 (48.1)	0.73	95 (59.4)	358 (47.9)	0.008
Ethnicity (%)						
Malay	132 (42.9)	1077 (51.4)	0.005	67 (41.9)	319 (42.7)	0.86
Indian	176 (57.1)	1018 (48.6)		93 (58.1)	429 (57.4)	
Education level (%)						
Primary/below	178 (57.8)	1138 (54.5)	0.27	94 (58.8)	473 (63.4)	0.27
Secondary/above	130 (42.2)	952 (45.6)		66 (41.3)	273 (36.6)	
Income (%)						
Income < S$2000	228 (74.8)	1549 (75.1)	0.89	134 (84.8)	570 (77.5)	0.040
Income ≥ S$2000	77 (25.3)	513 (24.9)		24 (15.2)	166 (22.6)	
Current smoking (%)	40 (13.0)	381 (18.2)	0.024	30 (18.8)	97 (13.0)	0.06
Diabetes duration (years)				8.26 (6.53)	7.32 (6.68)	0.19
Family history of diabetes (%)	167 (54.2)	876 (41.8)	<0.001	105 (66.0)	463 (62.0)	0.34
Use of anti-diabetes medication (%)				98 (61.3)	394 (52.7)	0.048
Use of anti-hypertensive medication (%)	96 (31.2)	447 (21.3)	<0.001	63 (39.9)	362 (48.7)	0.045
HbA_1c_ (%)	5.98 (0.33)	5.65 (0.35)	<0.001	8.74 (2.00)	7.28 (1.34)	<0.001
Total cholesterol (mmol/L)	5.48 (1.12)	5.53 (1.02)	0.46	5.27 (1.14)	5.17 (1.13)	0.28
HDL cholesterol (mmol/L)	1.14 (0.31)	1.25 (0.36)	<0.001	1.10 (0.32)	1.11 (0.30)	0.62
LDL cholesterol (mmol/L)	3.54 (0.93)	3.55 (0.91)	0.79	3.31 (1.00)	3.24 (0.94)	0.40
Hypertension (%)	194 (63.4)	1064 (50.9)	<0.001	113 (70.6)	543 (72.9)	0.56
Systolic BP (mm Hg)	140.0 (20.5)	135.6 (21.2)	<0.001	142.5 (19.1)	140.9 (19.7)	0.33
Diastolic BP (mm Hg)	80.3 (11.7)	78.3 (10.4)	0.002	80.1 (9.4)	78.4 (10.1)	0.06
BMI (kg/m^2^)	28.0 (4.8)	25.6 (4.3)	<0.001	26.5 (4.3)	28.1 (4.9)	<0.001
BMI Categories (kg/m^2^) (%)						
Underweight/normal (BMI < 25)	73 (23.7)	1018 (48.6)	<0.001	65 (40.6)	203 (27.1)	0.001
Overweight (25 ≤ BMI < 30)	153 (49.7)	778 (37.1)		67 (41.9)	336 (44.9)	
Obese (BMI ≥ 30)	82 (26.6)	299 (14.3)		28 (17.5)	209 (27.9)	

Data presented are mean (standard deviation) or frequency (percentage), where appropriate

*BMI* body mass index, *BP* blood pressure, *HbA*_*1c*_ glycated haemoglobin, *HDL* high density lipoprotein, *LDL* low density lipoprotein

*P-value was based on chi-square or *t*-test where appropriate

**Fig. 2:**
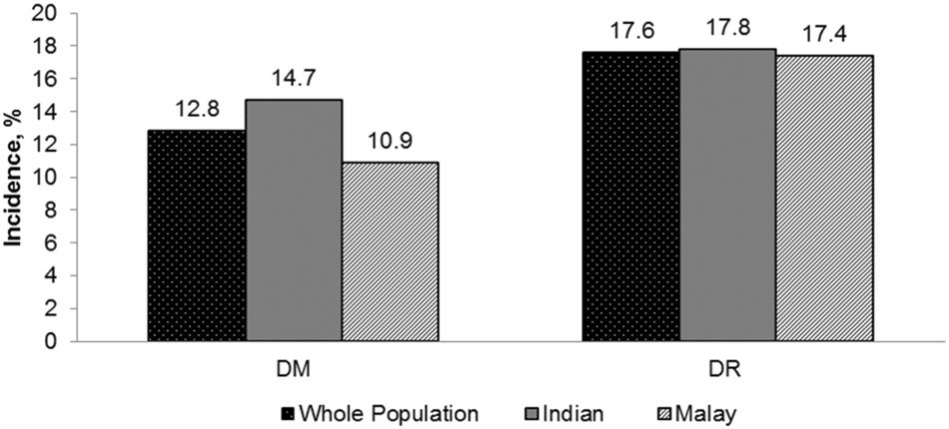
Incidence of DM and DR in the whole population and within ethnic groups

Over a median follow-up period of 6.2 years, incidence of DM was 12.8% in the overall population, and 10.9 and 14.7% in the Malay and Indian populations respectively (*p* = 0.001); incidence of DR was 17.6% in the overall population, and 17.4 and 17.8% in the Malay and Indian populations respectively (Fig. 2). There was a significant difference in the incidence of DM between Indians and Malays but no significant difference was observed with regards to incidence of DR.

Table 2 shows the associations between baseline BMI and incident DM. The incidence of DM was significantly higher in overweight and obese subjects than in subjects with BMI < 25 kg/m^2^. The RR of incident DM was higher in both the overweight (1.77) and obese (2.01) groups and also per SD increase in BMI in multivariable analysis after adjusting for potential confounders. In stratified analysis, the positive associations between overweight, obesity and incident DM remained significant across ethnic and gender subgroups without any significant interactions (*P* values of the interaction terms of BMI*gender and BMI*ethnicity were 0.87 and 0.15 respectively).

**Table 2:** Association of BMI with incident DM in the whole population and within subgroups of gender and ethnicity

	*N*	Incident DM *n* (%)	Incident diabetes			
			Age, gender, ethnicity adjusted RR (95% CI)	*P*-value	Multivariable adjusted RR (95% CI)^a^	*P*-value
Whole population	2403	308 (12.8)	1.53 (1.41, 1.65)		1.22 (1.12, 1.33)	
BMI (kg/m^2^) categories						
Underweight/normal (BMI < 25)	1091	73 (6.7)	Reference		Reference	
Overweight (25 ≤ BMI < 30)	931	153 (16.4)	2.47 (1.90, 3.21)	<0.001	1.77 (1.36, 2.29)	<0.001
Obese (BMI ≥ 30)	381	82 (21.5)	3.43 (2.54, 4.62)	<0.001	2.01 (1.50, 2.71)	<0.001
* P* trend				<0.001		<0.001
BMI per increase in SD (SD = 4.43)	2403	308 (12.8)	1.53 (1.41, 1.65)		1.22 (1.12, 1.33)	
Malays						
Underweight/normal (BMI < 25)	531	29 (5.5)	Reference		Reference	
Overweight (25 ≤ BMI < 30)	455	55 (12.1)	2.22 (1.44, 3.43)	<0.001	1.53 (1.00, 2.33)	0.049
Obese (BMI ≥ 30)	223	48 (21.5)	4.07 (2.56, 6.46)	<0.001	2.05 (1.28,3.28)	0.003
* P* trend				<0.001		0.002
BMI per increase in SD (SD = 4.72)	1209	132 (10.9)	1.52 (1.36, 1.71)		1.20 (1.05, 1.36)	
Indians						
Underweight/normal (BMI < 25)	560	44 (7.9)	Reference		Reference	
Overweight (25 ≤ BMI < 30)	476	98 (20.6)	2.67 (1.91, 3.72)	<0.001	1.97 (1.42, 2.73)	<0.001
Obese (BMI ≥ 30)	158	34 (21.5)	2.85 (1.90, 4.29)	<0.001	1.85 (1.24, 2.75)	0.002
* P* trend				<0.001		<0.001
BMI per increase in SD (SD = 4.12)	1194	176 (14.7)	1.53 (1.37, 1.70)		1.22 (1.10, 1.37)	
Men						
Underweight/normal (BMI < 25)	599	43 (7.2)	Reference		Reference	
Overweight (25 ≤ BMI < 30)	453	77 (17.0)	2.39 (1.68, 3.39)	<0.001	1.86 (1.30, 2.65)	0.001
Obese (BMI ≥ 30)	101	25 (24.8)	3.51 (2.24, 5.51)	<0.001	2.02 (1.28, 3.20)	0.003
* P* trend				<0.001		<0.001
BMI per increase in SD (SD = 3.80)	1153	145 (12.6)	1.59 (1.40, 1.81)		1.23 (1.07, 1.41)	
Women						
Underweight/normal (BMI < 25)	492	30 (6.1)	Reference		Reference	
Overweight (25 ≤ BMI < 30)	478	76 (15.9)	2.62 (1.75, 3.92)	<0.001	1.74 (1.18, 2.56)	0.005
Obese (BMI ≥ 30)	280	57 (20.4)	3.48 (2.28, 5.31)	<0.001	1.98 (1.30, 3.00)	0.001
* P* trend				<0.001		0.001
BMI per sd ↑, (SD = 4.81)	1250	163 (13.0)	1.50 (1.35, 1.65)		1.21 (1.09, 1.35)	

^a^Model adjusted for age, gender, ethnicity, family history of diabetes, income, education, current smoking status, systolic blood pressure, HbA_1c_, total cholesterol, HDL cholesterol

*P*-value for BMI × gender interaction = 0.87

*P*-value for BMI × ethnicity interaction = 0.15

Table 3 shows the associations between BMI and incident DR among persons with DM at baseline. The incidence of DR in the overweight and obese subjects was significantly lower than in subjects with BMI < 25 kg/m^2^. Obesity was significantly associated with DR in both age, sex, ethnicity-adjusted and the multivariable models. Overweight, while associated with DR in the first model, the association lost significance in the multivariable model. The inverse association between BMI and incident DR persisted when BMI was analysed as a continuous variable. In subgroup analysis by gender, the association between higher BMI and lower risk of DR was significant in women; in men, although the association was no longer statistically significant, the association remained consistent in direction (inverse). After stratification by ethnicity, in both Malay and Indian groups, the associations between higher BMI (as a categorical or continuous variable) and lower risk of incident DR remained consistent in direction, although no longer statistically significant. In analyses using incident VTDR as an outcome (*n* = 50), no significant associations were observed between BMI (as either a categorical or continuous variable) and VTDR (Table 4).

**Table 3:** Associations of BMI with incident DR in the whole population and stratified by gender and ethnicity

	*N*	Incident DR *n* (%)	Age, gender, ethnicity adjusted RR (95% CI)	Incident DR		*P*-value
				*P*-value	Multivariable adjusted RR (95% CI)^a^	
Whole population						
BMI (kg/m^2^) categories						
Underweight/normal (BMI < 25)	268	65 (24.3)	Reference		Reference	
Overweight (25 ≤ BMI < 30)	403	67 (16.6)	0.67 (0.50, 0.91)	0.011	0.80 (0.59, 1.09)	0.16
Obese (BMI ≥ 30)	237	28 (11.8)	0.49 (0.32, 0.74)	0.001	0.60 (0.39, 0.92)	0.018
* P* trend				<0.001		0.015
BMI per increase in SD (SD = 4.82)	908	160 (17.6)	0.74 (0.62, 0.87)		0.78 (0.66, 0.92)	
Malays						
Underweight/normal (BMI < 25)	108	28 (25.9)	Reference		Reference	
Overweight (25 ≤ BMI < 30)	164	26 (15.9)	0.59 (0.36, 0.97)	0.036	0.85 (0.52, 1.39)	0.52
Obese (BMI ≥ 30)	114	13 (11.4)	0.42 (0.23, 0.79)	0.007	0.60 (0.30, 1.22)	0.16
* P* trend				0.005		0.16
BMI per increase in SD (SD = 4.85)	386	67 (17.4)	0.74 (0.56, 0.97)		0.82 (0.62, 1.07)	
Indians						
Underweight/normal (BMI < 25)	160	37 (23.1)	Reference		Reference	
Overweight (25 ≤ BMI < 30)	239	41 (17.2)	0.74 (0.50, 1.09)	0.13	0.81 (0.53, 1.22)	0.31
Obese (BMI ≥ 30)	123	15 (12.2)	0.55 (0.32, 0.96)	0.036	0.61 (0.35, 1.07)	0.08
* P* trend				0.026		0.08
BMI per increase in SD (SD = 4.79)	522	93 (17.8)	0.74 (0.60, 0.91)		0.77 (0.62, 0.96)	
Men						
Underweight/normal (BMI < 25)	161	41 (25.5)	Reference		Reference	
Overweight (25 ≤ BMI < 30)	212	44 (20.8)	0.79 (0.55, 1.14)	0.21	0.93 (0.65, 1.35)	0.71
Obese (BMI ≥ 30)	80	10 (12.5)	0.45 (0.24, 0.86)	0.016	0.60 (0.32, 1.14)	0.12
*P* trend				0.009		0.13
BMI per increase in SD (SD = 4.21)	453	95 (21.0)	0.74 (0.59, 0.94)		0.82 (0.65, 1.04)	
Women						
Underweight/normal (BMI < 25)	107	24 (22.4)	Reference		Reference	
Overweight (25 ≤ BMI < 30)	191	23 (12.0)	0.51 (0.30, 0.86)	0.012	0.62 (0.37, 1.05)	0.073
Obese (BMI ≥ 30)	157	18 (11.5)	0.47 (0.27, 0.82)	0.008	0.51 (0.28, 0.92)	0.025
* P* trend				0.013		0.029
BMI per increase in SD (SD = 5.17)	455	65 (14.3)	0.73 (0.57, 0.93)		0.71 (0.56, 0.92)	

^a^Model adjusted for age, gender, ethnicity, family history of diabetes, income, education, current smoking status, systolic blood pressure, HbA_1c_, total cholesterol, HDL cholesterol, diabetes duration

*P*-value for BMI × gender interaction = 0.66

*P*-value for BMI × ethnicity interaction = 0.98

**Table 4:** Associations of BMI with Incident VTDR in the whole population

	*N*	Incident VTDR *n* (%)	Incident VTDR			
			Age, gender, ethnicity adjusted RR (95% CI)	*P*-value	Multivariable adjusted RR (95% CI)^a^	*P*-value
BMI (kg/m^2^) categories						
Underweight /Normal (BMI < 25)	389	23 (5.9)	Reference		Reference	
Overweight (25 ≤ BMI < 30)	518	16 (3.1)	0.52 (0.28, 0.98)	0.04	0.77 (0.35, 1.69)	0.51
Obese (BMI ≥ 30)	297	11 (3.7)	0.62 (0.28,1.34)	0.22	1.07 (0.38, 2.98)	0.90
*P* trend				0.16		0.92
BMI per increase in SD (SD = 4.78)	1204	50 (4.2)	0.68 (0.47, 0.98)		0.77 (0.45, 1.34)	

^†^Model adjusted for age, gender, ethnicity, family history of diabetes, income, education, current smoking status, systolic blood pressure, HbA_1c_, total cholesterol, HDL cholesterol and diabetes duration

In supplementary analyses, we repeated the analyses in Tables 2 and 3 1) excluding underweight participants (*n* = 79 and 5 respectively), 2) using BMI cut-points for public health action in Asians, recommended by WHO^9^. In these two independent analyses, the associations between BMI (as a continuous or as categorical variables) and incident DM remained statistically significant and consistent similar to the main analyses in Table 2. Furthermore, compared to normal, being underweight did not significantly affect the risk of incident DM or DR.

## Discussion

In this population-based cohort of Asian adults of Malay and Indian ethnicities, we showed that higher BMI was associated with an increased incidence of DM, but lower incidence of DR, independent of potential cofounders such as age, gender, ethnicity, duration of DM and HbA_1c_. These associations were consistent when BMI was analysed either as a categorical or continuous variable. In addition, the associations were consistent in analyses stratified by gender and ethnicity.

Our results for the positive association between increased BMI and incident DM are consistent with that of previous prospective studies, mostly in Western populations^7, 30^. The reasons for the positive association between BMI and DM have been well described and include insulin resistance and progressive beta cell dysfunction^31^. Three mechanisms linking obesity to insulin resistance include: (1) increased production of cytokines, including tumour necrosis factor-α, resistin and retinol-binding protein 4, leading to reduced levels of adiponectin and subsequent insulin resistance;^32^ (2) ectopic fat deposition in the liver and in the skeletal muscle;^33^ and (3) mitochondrial dysfunction leading to decreased insulin sensitivity. Some studies have also hypothesised a similar underlying defect whereby obesity induced cellular damage activate macrophages which worsens tissue inflammation leading to the pathogenesis of insulin resistance in the liver and peripheral tissues and resulting in damage to the β-cells^34, 35^. Hence, higher BMI can lead to an increased incidence of DM due to insulin resistance and β-cell dysfunction.

In the current study, we found an inverse association between obesity and incident DR and also between BMI (continuous) and DR. Our findings are similar to a number of cross-sectional Asian studies showing a protective association between overweight/obesity and DR^13–17, 19^. For instance, in the Beijing Community Diabetic Study, the Chennai Urban Rural Epidemiology Study and in a study of Asian Indian, Chinese and Creole Mauritians, higher BMI was associated with lower prevalence of DR, with the reported OR ranging from 0.50 to 0.95 in these studies^13, 16, 19^. Contrary to our study findings, most of the prospective studies done in Western populations had shown a positive association between higher BMI and DR^8, 12, 20^. For instance, the Diabetes Control and Complications Trial reported that higher BMI was significantly associated with incidence of DR (OR 1.11 (1.01–1.24)) among patients with type 1 DM^20^. In another study in Australia, higher BMI was significantly associated with any DR (OR 1.06, 1.01–1.11)^8^. To the best of our knowledge, there have been no studies examining the effect of BMI on the incidence of DR in Asian populations. Thus, it appears that the association of BMI and DR may differ between Asian and Western populations. In the current study, although overweight category showed a lower OR for incident DR similar to obesity category, the association lacked significance (*p* = 0.16). This could be due to the smaller number of cases in the overweight category. However, the *p*-trend across categories was significant (*p*-trend = 0.02) and in analyses using BMI as a continuous variable, a similar significant inverse association was found.

The exact mechanisms underlying the inverse association between BMI and DR in these Asian populations are uncertain. It has been shown that for the same BMI, body fat among Asians is higher by 3–5% as compared to Westerners^36^ in other words, for the same BMI, Asians and Westerners may have different levels of adiposity. However, even after adjusting for this by using the lower WHO Asian BMI cut-off values, our results still showed higher BMI was associated with lower incidences of DR. This inverse association ties in with the “obesity paradox”, which describes the trend of higher BMI being associated with better outcomes after percutaneous coronary intervention, as well as in conditions such as chronic kidney disease, congestive cardiac failure, peripheral arterial disease, stroke risk and thromboembolism^37–40^. Alternatively, it may not be that higher BMI is protective towards DR, but that individuals with lower BMI may have more severe DM (as patients with decompensated disease may undergo a catabolic phase resulting in unintentional weight loss) and thus have a higher risk of developing DR. Generally, long-term diabetes duration is associated with a lesser capacity for insulin secretion and these participants tend to have a lower BMI as compared to participants with shorter diabetes duration^41^. This could also be an important factor explaining the inverse relationship between BMI and the incidence of DR.

Evidence linking BMI levels with VTDR, has thus far been inconclusive, with some studies showing higher risk of VTDR with lower BMI^42^, and others with higher BMI^43^. In our study, no significant association was found between BMI and the risk of developing VTDR despite the incidence of VTDR being lower in the overweight (3.1%) and obese (3.7%) groups compared to those with BMI < 25 (5.9%). The reason for this is unclear, although our analyses were limited by a small number of cases of VTDR (*n* = 50). Because of the smaller number of events, we were also unable to examine DME and PDR separately.

Despite our findings that higher BMI is associated with lower risk of developing DR, caution must be made not to over-generalise that a higher BMI is preferable. Consistent with results of this study, it has been well reported that higher BMI is associated with increased risk of developing DM^7, 30^. Furthermore, and despite various studies suggesting a protective role of higher BMI in certain disease states as per the “obesity paradox”, it should be remembered that obesity has been associated with many health risks, as well as overall increased mortality^44^.

The strengths of this study include a large sample size of two different ethnicities; longitudinal data over a 6-year period with a relatively low drop-out rate; and a standardised and comprehensive protocol for grading DR and assessing risk factors. One of our limitations arises from BMI being an imperfect measurement of body fat and composition, with more accurate alternative measures such as waist hip ratio to measure central abdominal obesity having been described^15^. Furthermore, this being an epidemiological study involving a large population, we only had baseline and follow-up HbA1c measurements and hence were unable to use the mean of serial HbA1c measurement throughout the 6-year period. Additionally, we were not able to adjust for the effects of other potential confounders such as diet and physical activity as such data were not collected.

In conclusion, we showed in this large cohort of Asian Malays and Indians, that higher BMI was associated with a higher incidence of DM but a lower incidence of DR over a 6-year period. As body weight and DR are complex traits influenced by numerous environmental and genetic factors, further genetic studies may be warranted to investigate the different effects of BMI on the risk of developing DR in Asian versus Western populations.

## References

[1] Leasher JL (2016). Global estimates on the number of people blind or visually impaired by diabetic retinopathy: a meta-analysis from 1990 to 2010. Diabetes Care.

[2] Wong TY, Cheung CM, Larsen M, Sharma S, Simo R (2016). Diabetic retinopathy. Nat. Rev. Dis. Prim..

[3] Cheung N, Mitchell P, Wong TY (2010). Diabetic retinopathy. Lancet.

[4] Ding J, Wong TY (2012). Current epidemiology of diabetic retinopathy and diabetic macular edema. Curr. Diab Rep..

[5] Wong TY (2008). Relation between fasting glucose and retinopathy for diagnosis of diabetes: three population-based cross-sectional studies. Lancet.

[6] Yau JW (2012). Global prevalence and major risk factors of diabetic retinopathy. Diabetes Care.

[7] Nguyen NT, Nguyen XM, Lane J, Wang P (2011). Relationship between obesity and diabetes in a US adult population: findings from the National Health and Nutrition Examination Survey, 1999-2006. Obes. Surg..

[8] Dirani M (2011). Are obesity and anthropometry risk factors for diabetic retinopathy? The diabetes management project. Invest Ophthalmol. Vis. Sci..

[9] Consultation WHOE. (2004). Appropriate body-mass index for Asian populations and its implications for policy and intervention strategies. Lancet.

[10] Deurenberg-Yap M, Chew SK, Deurenberg P (2002). Elevated body fat percentage and cardiovascular risks at low body mass index levels among Singaporean Chinese, Malays and Indians. Obes. Rev..

[11] Rush EC, Freitas I, Plank LD (2009). Body size, body composition and fat distribution: comparative analysis of European, Maori, Pacific Island and Asian Indian adults. Br. J. Nutr..

[12] Ballard DJ (1986). Risk factors for diabetic retinopathy: a population-based study in Rochester, Minnesota. Diabetes Care.

[13] Dowse GK (1998). Prevalence and risk factors for diabetic retinopathy in the multiethnic population of Mauritius. Am. J. Epidemiol..

[14] Lim LS (2010). C-reactive protein, body mass index, and diabetic retinopathy. Invest Ophthalmol. Vis. Sci..

[15] Man RE (2016). Differential association of generalized and abdominal obesity with diabetic retinopathy in Asian patients with type 2 diabetes. JAMA Ophthalmol..

[16] Rema M (2005). Prevalence of diabetic retinopathy in urban India: the Chennai Urban Rural Epidemiology Study (CURES) eye study, I. Invest Ophthalmol. Vis. Sci..

[17] Rooney D (2015). Body mass index and retinopathy in Asian populations with diabetes mellitus. Acta Diabetol..

[18] van Leiden HA (2003). Risk factors for incident retinopathy in a diabetic and nondiabetic population: the Hoorn study. Arch. Ophthalmol..

[19] Xu J (2012). Prevalence and risk factors for diabetic retinopathy: the Beijing Communities Diabetes Study 6. Retina.

[20] Zhang L, Krzentowski G, Albert A, Lefebvre PJ (2001). Risk of developing retinopathy in diabetes control and complications trial type 1 diabetic patients with good or poor metabolic control. Diabetes Care..

[21] Foong AW (2007). Rationale and methodology for a population-based study of eye diseases in Malay people: The Singapore Malay eye study (SiMES). Ophthalmic Epidemiol..

[22] Lavanya R (2009). Methodology of the Singapore Indian Chinese Cohort (SICC) eye study: quantifying ethnic variations in the epidemiology of eye diseases in Asians. Ophthalmic Epidemiol..

[23] Cheung CMG (2017). Six-year incidence of age-related macular degeneration in Asian Malays: The Singapore Malay Eye Study. Ophthalmology.

[24] Sabanayagam C (2017). Singapore Indian Eye Study 2: methodology and impact of migration on systemic and eye outcomes. Clin. Exp. Ophthalmol..

[25] Kramer H (2004). Racial/ethnic differences in hypertension and hypertension treatment and control in the multi-ethnic study of atherosclerosis (MESA). Am. J. Hypertens..

[26] Rosman M (2012). Singapore Malay Eye Study: rationale and methodology of 6-year follow-up study (SiMES-2). Clin. Exp. Ophthalmol..

[27] Wong TY (2008). Prevalence and risk factors for diabetic retinopathy: the Singapore Malay Eye Study. Ophthalmology.

[28] Group ETDRSR. (1991). Grading diabetic retinopathy from stereoscopic color fundus photographs--an extension of the modified Airlie House classification. ETDRS report number 10. Early Treatment Diabetic Retinopathy Study Research Group. Ophthalmology.

[29] Zou G (2004). A modified poisson regression approach to prospective studies with binary data. Am. J. Epidemiol..

[30] Narayan KM, Boyle JP, Thompson TJ, Gregg EW, Williamson DF (2007). Effect of BMI on lifetime risk for diabetes in the U.S. Diabetes Care.

[31] Eckel RH (2011). Obesity and type 2 diabetes: what can be unified and what needs to be individualized?. J. Clin. Endocrinol. Metab..

[32] Deng Y, Scherer PE (2010). Adipokines as novel biomarkers and regulators of the metabolic syndrome. Ann. N. Y. Acad. Sci..

[33] Larson-Meyer DE (2011). Intrahepatic and intramyocellular lipids are determinants of insulin resistance in prepubertal children. Diabetologia.

[34] Hotamisligil GS, Erbay E (2008). Nutrient sensing and inflammation in metabolic diseases. Nat. Rev. Immunol..

[35] Weisberg SP (2003). Obesity is associated with macrophage accumulation in adipose tissue. J. Clin. Invest..

[36] Deurenberg P, Deurenberg-Yap M, Guricci S (2002). Asians are different from Caucasians and from each other in their body mass index/body fat per cent relationship. Obes. Rev..

[37] Beddhu S, Pappas LM, Ramkumar N, Samore M (2003). Effects of body size and body composition on survival in hemodialysis patients. J. Am. Soc. Nephrol..

[38] Li W (2015). Body mass index and stroke risk among patients with type 2 diabetes mellitus. Stroke.

[39] Oreopoulos A (2008). Body mass index and mortality in heart failure: a meta-analysis. Am. Heart J..

[40] Romero-Corral A (2006). Association of bodyweight with total mortality and with cardiovascular events in coronary artery disease: a systematic review of cohort studies. Lancet.

[41] Gupta D, Krueger CB, Lastra G (2012). Over-nutrition, obesity and insulin resistance in the development of beta-cell dysfunction. Curr. Diabetes Rev..

[42] Klein R, Klein BE, Moss SE, Davis MD, DeMets DL (1984). The Wisconsin epidemiologic study of diabetic retinopathy. III. Prevalence and risk of diabetic retinopathy when age at diagnosis is 30 or more years. Arch. Ophthalmol..

[43] Ozmen B, Boyvada S (2003). The relationship between self-monitoring of blood glucose control and glycosylated haemoglobin in patients with type 2 diabetes with and without diabetic retinopathy. J. Diabetes Complicat..

[44] Calle EE, Rodriguez C, Walker-Thurmond K, Thun MJ (2003). Overweight, obesity, and mortality from cancer in a prospectively studied cohort of U.S. adults. N. Engl. J. Med..

